# Highly Accelerated Dual-Pose Medical Image Registration via Improved Differential Evolution

**DOI:** 10.3390/s25154604

**Published:** 2025-07-25

**Authors:** Dibin Zhou, Fengyuan Xing, Wenhao Liu, Fuchang Liu

**Affiliations:** School of Information Science and Technology, Hangzhou Normal University, Hangzhou 311121, China; dibinz@zju.edu.cn (D.Z.); xfyprude3918@163.com (F.X.); liufc@hznu.edu.cn (F.L.)

**Keywords:** dual-pose, 2D-3D image registration, differential evolutionary algorithm, composite similarity, digital reconstruction of radiographic images

## Abstract

Medical image registration is an indispensable preprocessing step to align medical images to a common coordinate system before in-depth analysis. The registration precision is critical to the following analysis. In addition to representative image features, the initial pose settings and multiple poses in images will significantly affect the registration precision, which is largely neglected in state-of-the-art works. To address this, the paper proposes a dual-pose medical image registration algorithm based on improved differential evolution. More specifically, the proposed algorithm defines a composite similarity measurement based on contour points and utilizes this measurement to calculate the similarity between frontal–lateral positional DRR (Digitally Reconstructed Radiograph) images and X-ray images. In order to ensure the accuracy of the registration algorithm in particular dimensions, the algorithm implements a dual-pose registration strategy. A PDE (Phased Differential Evolution) algorithm is proposed for iterative optimization, enhancing the optimization algorithm’s ability to globally search in low-dimensional space, aiding in the discovery of global optimal solutions. Extensive experimental results demonstrate that the proposed algorithm provides more accurate similarity metrics compared to conventional registration algorithms; the dual-pose registration strategy largely reduces errors in specific dimensions, resulting in reductions of 67.04% and 71.84%, respectively, in rotation and translation errors. Additionally, the algorithm is more suitable for clinical applications due to its lower complexity.

## 1. Introduction

With the continuous improvement of medical standards, medical imaging technology plays an increasingly important role in modern clinical medicine [1]. It has become an indispensable component of clinical diagnosis, surgical planning, and condition assessment, among other application areas. Images from different modalities provide diverse diagnostic support and functional information, and a single image does not offer sufficient data for comprehensive analysis [2]. To enhance the quality of medical diagnosis and treatment, it is typically necessary to comprehensively analyze patients’ image data from multiple modalities during clinical treatment. However, relying solely on doctors’ experience to analyze medical images from different modalities is challenging. The use of medical image registration technology addresses the problem of multimodal medical image analysis by mapping image information from multiple modalities to the optimal spatial correspondence through optimal geometric transformations. Medical image registration serves as the foundation and prerequisite for medical imagery fusion, holding significant value in both medical research and practical clinical applications [3,4,5].

Numerous medical image registration techniques have been proposed by domestic and international scholars to enhance the accuracy of 2D-3D medical image registration. These algorithms can be broadly categorized into two groups: those based on image features and those relying on image grayscale [6]. To accurately locate and track the patient’s lesions [7], feature-based methods typically depend on internal inherent features and markers that are either implanted in the patient’s body or applied to the skin’s surface before surgery [8]. Regodić M et al. [9] achieved image registration by automatically identifying pairs of skin-attached marker points to expedite the registration process. However, this approach falls short of meeting minimally invasive requirements. Yu et al. [10] proposed using a B-spline statistical transformation model to extract image features for implementing a non-rigid 2D-3D registration algorithm. Nevertheless, the model requires retraining to apply the algorithm to other sections, and the operational procedure is somewhat intricate. In general, feature-based methods do not necessitate highly accurate registration algorithms [11]. Techniques like training models or markers can be employed to retrieve spatial feature information of the lesion [12]. However, this approach is more labor-intensive in the feature extraction step, requires additional calibration equipment, and may pose risks to the patient’s body [13].

Ongoing research is focusing on medical image registration techniques based on image grayscale to address the requirements of clinical medical treatments [14]. To achieve optimal registration, the fundamental approach involves initially acquiring 2D images using Digital Reconstructed Radiography (DRR) technology. Subsequently, an optimization strategy is selected, and continuous iterations are performed to minimize the similarity difference between the reconstructed image and the X-ray image [15]. Among these, various similarity measures have been proposed. These encompass the grayscale-based SSD (Sum of Squared Differences) algorithm, the NCC (Normalized Cross Correlation) similarity algorithm, and approaches based on mutual information. Notably, recent years have witnessed the emergence of effective solutions facilitated by deep learning-based algorithms [16]. Siamese neural networks [17,18] belong to the category of few-shot learning networks, primarily achieving the goal of image matching by measuring the similarity between two input vectors. McCauley J et al. [19] suggested a FaceNet to quantify the facial similarity of identical twins in general. A deep graph similarity learning model SimGNN is proposed in Bai et al. [20], which also aims to learn similarity for chemical compounds as one of the tasks. Nonetheless, deep learning models necessitate vast volumes of labeled data for training. Insufficient data could make it difficult for these models to effectively generalize to new examples. The processing of extensive medical datasets is also a time-consuming task.

In single-modality medical image registration, the grayscale-based method has demonstrated promising results [21]. However, in certain scenarios, relying on a single global gray value for the similarity measure may overlook crucial image details such as bone structures and muscle tissues. This can result in an algorithm lacking robustness and being susceptible to noise interference. The limitation of this method lies in its sensitivity to grayscale variations, presenting a significant challenge when dealing with multimodal or fuzzy medical images. In line with research on grayscale-based registration methods, a majority of studies focus on single-plane registration techniques. Banks et al. [22] simulated elements such as light sources and projection planes on a computer for registration, utilizing contour information to detect similarity. Although this technique yields favorable registration results, the error in the Z-axis is considerably larger than that in the X-axis and Y-axis. Investigation into the root cause of this issue reveals that the contour information extracted by the single posture method is missing relevant data in the Z-axis direction.

To address the aforementioned challenges, this paper introduces a dual-pose medical image registration algorithm grounded in enhanced differential evolution. The primary contributions of this research can be encapsulated as follows.

(1) Dual-Pose Strategy for Dimensional Accuracy: To tackle the issue of significant errors in specific dimensions, we introduce a dual-pose strategy for medical image registration. Utilizing the prior DRR image from the frontal pose, which is derived from the single-posture image, we generate the DRR image for the lateral pose by amalgamating the transformation matrix employed for lateral pose conversion. This approach offers a novel perspective to validate the registration accuracy of the single-posture stance from a different viewpoint, effectively rectifying biases in specific dimensions.
(2) Composite Similarity Measure for Fuzzy Image Challenges: To mitigate the interference from fuzzy images during registration, we design a composite similarity measure. This measure aims to precisely compute the composite similarity between the frontal–lateral posture DRR image and the X-ray image using contour-based similarity metrics, ensuring accurate registration.
(3) Phased Differential Evolution (PDE) for Optimal Results: Addressing the propensity of the registration outcome to converge to local optima, we propose a Phased Differential Evolution (PDE) optimization algorithm. This iterative approach refines the objective function, continuously calculating the composite similarity between the frontal–lateral posture DRR image and the X-ray image to achieve optimal registration results.
(4) Efficiency Enhancement via GPU-Accelerated Parallel Computation: To expedite the registration process, we employ multi-threaded parallel computation leveraging Graphics Processing Units (GPUs). This significantly boosts the efficiency of DRR image generation and minimizes data transmission overheads during the registration procedure.

Detailed descriptions of the associated algorithms follow below.

## 2. Method

A 2D-3D medical image registration aims to determine an optimal spatial position match for the medical image (floating image) through spatial transformation operations. This match ensures the alignment of the floating image with the spatial coordinates of corresponding points on the reference image.

The medical image registration algorithm outlined in this paper comprises the following four main steps.

(1) Acquisition of Reference Image Data: In contrast to traditional single-posture registration algorithms, this step involves acquiring X-ray image information from two frontal–lateral positions.
(2) Generation of Frontal and Lateral DRR Images: After identifying the six-degree-of-freedom parameters of the initial pose through manual registration, DRR images for frontal and lateral poses are obtained. Utilizing the transformation matrix (*LateralMat*) for lateral pose projection, GPU parallel processing accelerates the rendering process to generate the DRR images.
(3) Similarity Measure Calculation: Contour information is extracted from both the reference image and the floating image. The composite similarity between the DRR image and the reference image under the dual-pose configuration is then calculated.
(4) Optimization Algorithm for Parameter Tuning: An optimization algorithm is employed to find the optimal parameters by identifying the smallest or largest value of the objective function.

**Figure 1:**
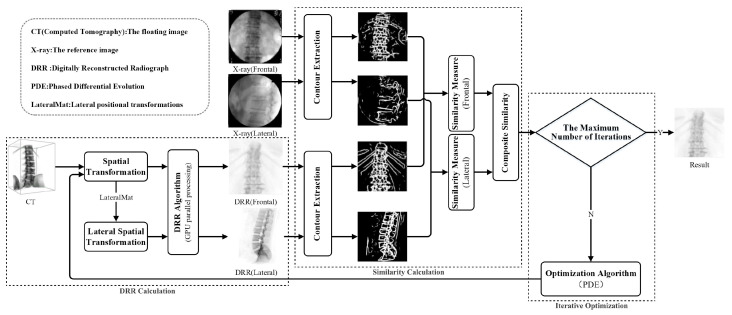
Dual-pose medical image registration process based on improved differential evolution.

The process of dual-pose medical image registration, based on the improved differential evolution, is illustrated in Figure 1.

## 3. A Dual-Pose Medical Image Registration Algorithm Based on Improved Differential Evolution

### 3.1. Acquisition of DRR in the Frontal and Lateral Positions

**Figure 2:**
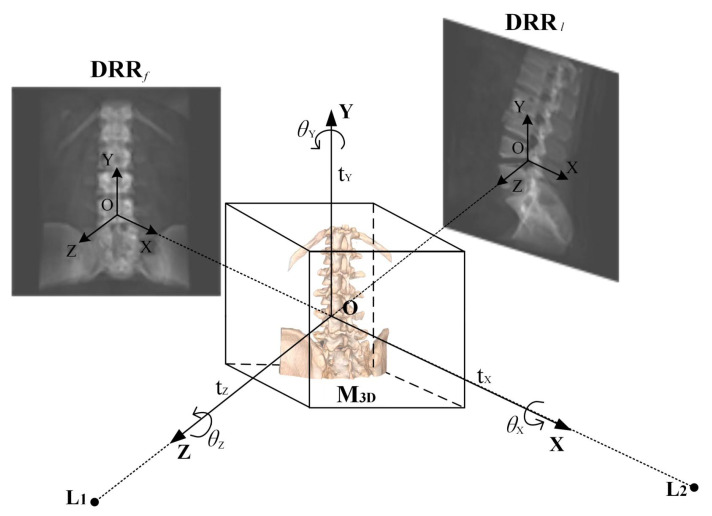
Obtain the dual-pose DRR image’s projection schematic diagram.

In conventional single-posture registration, a single planar image provides relatively limited information for medical image registration and proves ineffective in analyzing spatial information across all six degrees of freedom of the targeted body data [23,24]. The absence of corrective analysis for multiple postures results in significant registration errors, particularly in specific dimensions. Therefore, utilizing a dual-pose image as the reference image for registration, containing more comprehensive image and projection view information, enhances the potential for superior registration outcomes. The projection model is illustrated in Figure 2.

$L_{1}$ and $L_{2}$ represent simulated point light sources in the frontal and lateral positions, respectively. $M_{3D}$ denotes the CT data, and the transformation of the CT data is determined by the translation parameters $t_{X},t_{Y},t_{Z}$ along the X, Y, and Z axes of the spatial coordinate system and the rotation parameters $\theta_{X},\theta_{Y},\theta_{Z}$ around the respective axes. ${DRR}_{f}$ and ${DRR}_{l}$ are the frontal and lateral DRR images generated from the CT body data after the frontal and lateral position projections, respectively. When the CT body data undergo geometric transformations such as translation or rotation, the 2D image projected onto the projection plane undergoes corresponding transformations. In the registration task of CT images projected by the DRR (Digital Reconstructed Radiograph) and X-ray images, the core goal is to establish the spatial transformation relationship between them, so that the DRR projection can accurately map to the anatomical structures of the actual X-ray images. This process can be formalized as a parameter optimization problem: finding the optimal transformation parameter ${\left(T,R\right)}^{*}$ to maximize the similarity S(·,·) between the transformed DRR image and the X-ray image. The mathematical expression of this optimization problem is Equation (1).

(1) $$\begin{array}{cc} {\left(T,R\right)}^{*} & =\arg\max_{\left(T,R\right)}S\left(\mathrm{DRR}\left(T\left(t_{x},t_{y},t_{z}\right)\cdot R\left(\theta_{x},\theta_{y},\theta_{z}\right)\cdot \mathrm{CT}\right),I_{X-\mathrm{ray}}\right) \end{array}$$

In Equation (1), $T(t_{X},t_{Y},t_{Z})$ denotes a translation matrix, and the *Mat* contains a rotation matrix describing the relationship of rotation and a translation vector describing the relationship of translation in the spatial transformation of the medical image. The transformation matrix (*FrontalMat*) is computed from the six-degree-of-freedom parameters of the frontal position as follows.

(2) $$\mathrm{FrontalMat}=\mathrm{Mat}(t_{X},t_{Y},t_{Z},\theta_{X},\theta_{Y},\theta_{Z})$$

In Equation (2), *Mat* is the method of calculating the matrix by Equation (1). And then *LateralMat*, the matrix of lateral positional transformations, is derived by Equation (3) based on the *TransMat*, the known matrix of frontal–lateral pose transformation from the image data. According to a matrix of frontal–lateral positional transformations, the DRR image of the frontal and lateral position is finally obtained.

(3) LateralMat=TransMat·FrontalMat

### 3.2. Digitally Reconstructed Radiograph Imaging Based on GPU Parallel Acceleration

**Figure 3:**
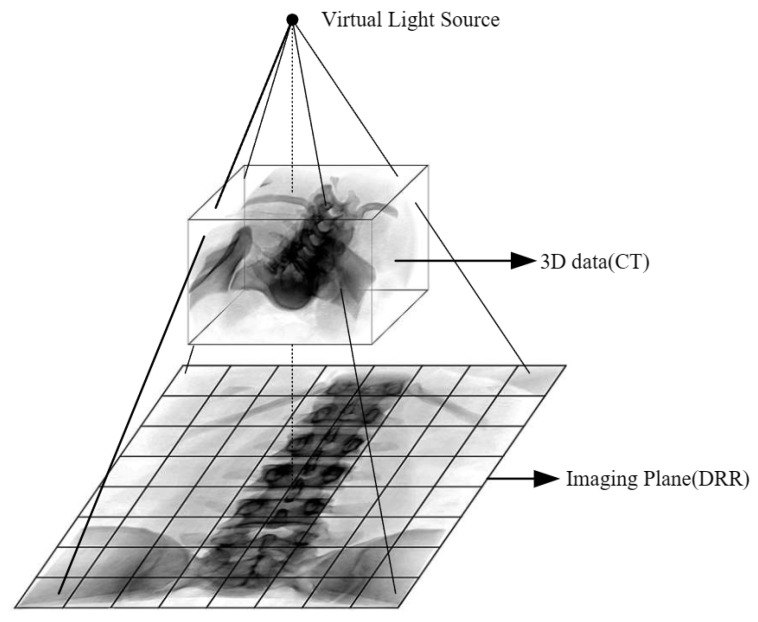
Schematic diagram of ray projection algorithm.

The Digitally Reconstructed Radiograph (DRR) [25] involves processing CT data through analog projection rendering to generate virtual X-ray 2D images, facilitating the conversion from 3D data to 2D images. Specifically, the core process of the DRR algorithm includes the following steps: First, the 3D CT volume data are transformed into the perspective of a virtual camera, defining the projection center, detector position, and display range. Then, virtual X-rays are emitted from each pixel of the detector, uniformly sampling the CT data along the ray direction (trilinear interpolation is commonly used to improve accuracy) and accumulating the attenuation values of points along the way. Finally, these accumulated values are converted into grayscale to generate a 2D projection image. This process is illustrated in Figure 3.

Traditional DRR algorithms are typically implemented on the CPU, and the generation process consumes a significant amount of time due to the simulation of a large number of X-rays with limited parallelism. To address the need for real-time processing, Fluck et al. [26] introduced an accelerated DRR generation method based on GPU. This approach significantly enhances the efficiency of image generation.

**Figure 4:**
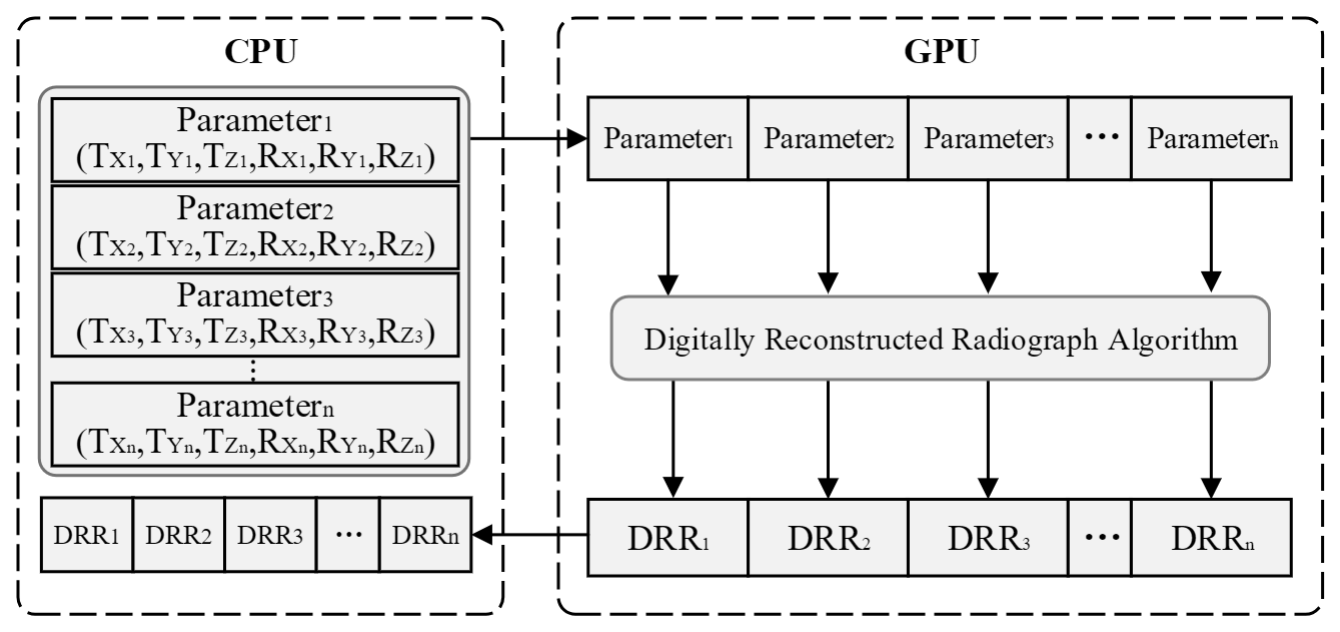
The Process for DRR calculating parallelly on GPU.

Since each simulated X-ray that passes through the CT data does not intersect each other and the CT accumulated value is calculated in an identical manner, It is consistent with the characteristics of the CUDA for parallel computation. Using the GPU for multi-threaded parallel computation to realize the simultaneous rendering of multiple simulated X-rays greatly improves the efficiency of DRR generation [27]. Efficiency is impacted by frequent read and write data operations between main memory and graphics memory in traditional algorithms. Every time a CUDA computation is triggered, there must be an extra GPU startup timing overhead. An algorithm for multiple DRR generation in paralleling is designed in order to address this problem. Multiple sets of pose parameters are input, the pose data are transferred to the graphics memory, and multiple DRR images are generated by parallel computation of CUDA corresponding to this set of poses. Finally, send these image data back to the main memory by sequential output. The process of parallel computation is shown in Figure 4. Multiple sets of pose data are transmitted simultaneously by the GPU-based parallel generation of the DRR algorithm at a time, which uses multiple threads to compute several DRR images parallelly. Compared to the conventional methods, this process significantly reduces the time overhead and improves the algorithm’s execution efficiency because it only requires one GPU startup.

GPU acceleration represents an effective strategy for improving system performance and expanding its applicability, particularly in real-time processing scenarios with stringent computational requirements. Nonetheless, the system also exhibits stable performance on CPU-only platforms in resource-limited settings, reflecting its strong generalizability and adaptability across diverse deployment environments.

### 3.3. Similarity Measure

The similarity measure for medical images quantifies the degree of resemblance between a reference image and a floating image [28], providing a metric for assessing their likeness. This measure proves invaluable in the analysis and registration of multimodal images or those acquired from different devices, playing a crucial role in disease tracking, surgical navigation, and treatment plan development [29]. Fuzzy medical images, often characterized by uncertainties such as noise and artifacts, pose challenges in extracting local information. These uncertainties can hinder the accurate measurement of the degree of match between images. To address these challenges, this paper introduces a similarity measure based on contour points. The algorithm unfolds in the following steps.

In the initial step, the obtained frontal–lateral position X-ray image and frontal–lateral position DRR image undergo preprocessing. Specifically, contour extraction images are computed for both the frontal–lateral pose DRR images and frontal–lateral pose X-ray images using the classical Canny operator [30]. The contour extraction process involves the computation of contour extraction images for the frontal–lateral positional DRR images and frontal–lateral positional X-ray images, each employing the classical Canny operator. The resulting contour-extracted images from the DRR and X-ray images serve as the reference and floating images, respectively. The coordinates of the contour points in these images are stored using the variable *S* as outlined below.

(4) $$S=\{S_{1},S_{2},\ldots ,S_{N}\}$$

In Equation (4), *N* represents the number of contour points. The search for contour points commences by defining a region centered on the corresponding pixel coordinate points in the floating image. The search is then conducted within this region. If a corresponding contour point is found within the region, a weight (*c*) is assigned based on the distance from the center, contributing to the matching score (*W*). The matching score for the i-th contour point is defined as Equation (5). The final similarity is calculated by summing the matching scores W of all contour points and normalizing by the number of points, as shown in Equation (6).

(5) $$s_{i}\left(d_{i}\right)=\left\{\begin{array}{cc} 1, & d_{i}=0\\ w_{1}, & 0<d_{i}\leq a\\ w_{2}, & a<d_{i}\leq b\\ 0, & d_{i}>b \end{array}\right.$$

(6) $$\mathrm{Similarity}=\frac{1}{N}\sum_{i=1}^{N}s_{i}\left(d_{i}\right)$$

After the algorithm searches all the contour points of the reference image, it finally obtains the similarity of the front position ${\mathrm{Sim}}_{f}$ and lateral position ${\mathrm{Sim}}_{l}$. The similarity measure, which fully considers the positional relation of the matched contour point pairs, derives a high similarity value if the difference in the edge point positions between the reference image and the floating image is small, and vice versa for a low similarity value. Using weighted calculations at different ranges from the central region improves algorithm accuracy and optimization efficiency. The algorithm is applied to medical images where the changes are not particularly significant and can effectively reflect the subtle changes between images. Algorithm 1 is represented as follows.
**Algorithm 1.** Similarity measure based on contour points.N: Number of contour point pixels$S_{i}^{r}$: Coordinates of the contour points of the reference image$S_{i}^{t}$: Coordinates of the contour points in the image to be registered1: Preprocessing of images to remove noise2: Computing image contour images using the Canny operator3: Calculating similarity:**for i = 1 : N do:**       if $S_{i}^{r}$=$S_{i}^{t}$           Calculate the number of contour points in the region counted in W           Construct $W_{i}=cW$           Construct $W_{d}+=W_{i}$**End(for)**       *Similarity*
$=\frac{W_{d}}{N}$Return: *Similarity*End

### 3.4. Composite Similarity

In traditional single-posture medical image registration, a single similarity metric is typically employed to assess the degree of matching between the reference image and the floating image. This approach encounters challenges in effectively handling multimodal medical images. Moreover, it exhibits high sensitivity to noise and artifacts in fuzzy images, potentially compromising the accuracy of similarity measurements and leading to suboptimal registration outcomes. To enhance the overall accuracy of image similarity assessment, particularly in the context of the dual-pose strategy, this paper introduces the concept of composite similarity—a composite measure designed to comprehensively evaluate similarity. This involves assigning weighting factors, denoted as a and b. The composite similarity is derived by assigning weights to the similarity scores calculated for the frontal and lateral positions in the preceding step, as illustrated in Equation (7).

(7) $$Sim=a\cdot {\mathrm{Sim}}_{f}+b\cdot {\mathrm{Sim}}_{l}$$

The weight factors *a*, *b* ∈ (0,1) in the equation, should be chosen to appropriately emphasize the significance of different similarity measures in determining the final similarity. Recognizing that the positive pose image holds greater importance for similarity measurement in medical image registration, whereas the lateral posture image serves as more of an auxiliary guide, contributing slightly less to the similarity measure than the positive pose image, it is established that *a* > *b*. Incorporating the composite similarity during the iterations guides the adjustment of positional parameters toward a trend that is more conducive to achieving accurate registration results, thereby enhancing the reliability of the image similarity assessment.

### 3.5. Intelligent Optimization Algorithm

Utilizing the aforementioned composite similarity, the process of medical image registration is conceptualized as solving a multi-parameter optimization problem. This involves iteratively calculating and refining the optimal solution for the parameters through an optimization search method. The objective function of the optimization algorithm can be expressed as follows.

(8) $$S_{p}=\arg\underset{S_{p}}{Max}\left[a\cdot F_{f}\left(R_{f},{DRR}_{f_{S_{p}}}\right)+b\cdot F_{l}\left(R_{l},{DRR}_{l_{S_{p}}}\right)\right]$$

In Equation (8), $S_{p}$ represents the spatial transformation of the CT data using DRR technology, encompassing the parameters of the objective function ($t_{X},t_{Y},t_{Z},{`}_{X},{`}_{Y},{`}_{Z}$). $R_{f},R_{l}$ denote reference images of the frontal–lateral postures, respectively. ${DRR}_{f}$ and ${DRR}_{l}$ refer to the floating images of the frontal–lateral postures under transformation ($S_{p}$), respectively. $F_{f},F_{l}$ represent the similarity measures ${\mathrm{Sim}}_{f}$ and ${\mathrm{Sim}}_{l}$ used in calculating the contours of two sets of frontal and lateral images.

**Figure 5:**
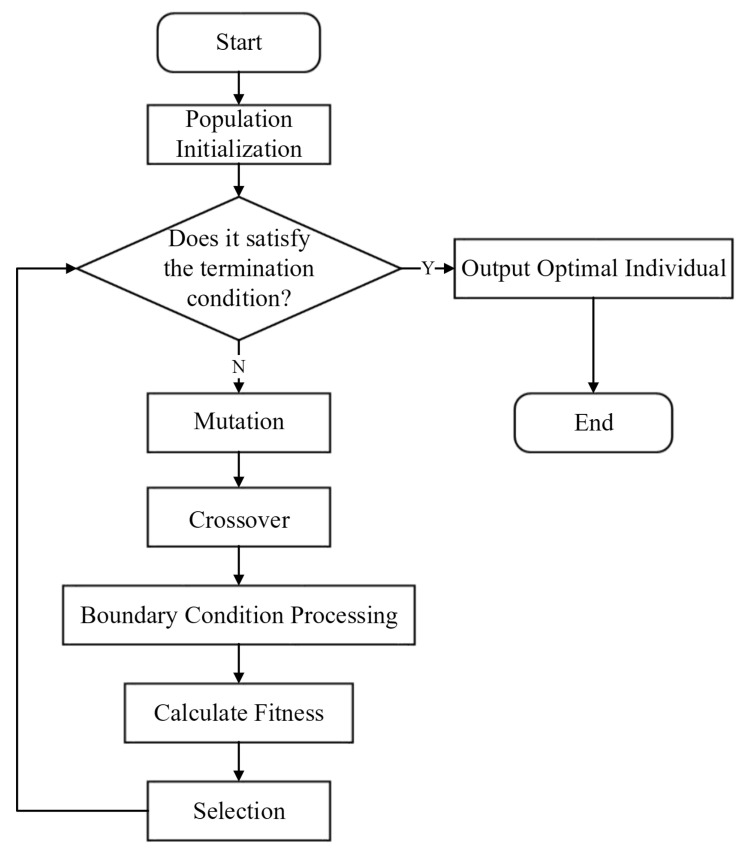
Image/Differential evolution algorithm process.

This paper employs Phased Differential Evolution (PDE) as the optimization algorithm for iterative optimization, aiming to find the optimal pose parameters. Compared to other optimization algorithms, the PDE algorithm has a greater capability to converge toward precise solutions and exhibits stronger global search abilities. Throughout the iterative process, PDE often demonstrates rapid convergence speed and is less prone to becoming trapped in local optima. Therefore, the PDE algorithm is highly suitable for the search task of target skeleton image registration. Simulating the principles of crossover and mutation in genetics, the differential evolution algorithm is designed using genetic operators [31]. The standard process of the differential evolution algorithm is illustrated in Figure 5.

#### 3.5.1. Population Initialization

Randomly initialize D-dimensional parameter vectors of quantity *NP* as *X*, where each set of parameters represents

(9) $$X_{i,G}=\left\{x_{i,G}^{1},\ldots ,x_{i,G}^{D}\right\},\;i=1,\ldots ,\mathrm{NP}$$

The number of solutions, *NP*, is chosen according to the circumstances. In the process of medical image registration, each generated DRR image represents a set of pose parameters in the optimization algorithm. In this paper, *NP* is selected as 10, where each set of pose parameters requires six degrees of freedom. Thus, the dimension *D* is chosen as 6.

#### 3.5.2. Mutation

Once initialized, for each particle within the population, a specific mutation strategy can be employed to generate the associated mutation vector $V_{i,G}$. The mutation operation in the PDE algorithm is a crucial component, where mutation is regarded as a stochastic element of change or disturbance. The mutation vector in the PDE algorithm is generated from parent differential vectors and combined with the parent individual vectors to create new individual vectors through crossover. To maintain population diversity, the following mutation strategy “*DE/rand/2*” [32] is employed, which utilizes three randomly selected individual vectors to generate the mutation vector, as depicted in Equation (10).

(10) $$V_{i,G}=X_{r_{1}^{i},G}+F\cdot \left(X_{r_{2}^{i},G}-X_{r_{3}^{i},G}\right)+F\cdot \left(X_{r_{4}^{i},G}-X_{r_{5}^{i},G}\right)$$

In the Equation (10), the indices $r_{1}^{i},r_{2}^{i},r_{3}^{i},r_{4}^{i},r_{5}^{i}$ are mutually exclusive integers randomly generated within the range [1, *NP*]. For each mutation vector, these indices are randomly generated once. The scaling factor *F* is a control parameter for scaling the differential vectors. If *F* is excessively small, it reduces the population diversity, leading to premature convergence of the algorithm. Conversely, if *F* is excessively large, it decreases the algorithm’s convergence speed and reduces the accuracy of obtaining the global optimum solution. During the initial stages of evolution, due to the considerable diversity among individuals in the population, the differential vectors used as perturbations in mutation are also large. This significant perturbation among individuals favors global exploration in the algorithm. As the evolution progresses and the algorithm approaches convergence, the differences among individuals in the population decrease. Consequently, the differential vectors used as perturbations in mutation also adaptively reduce in size. This adaptive reduction in perturbation is beneficial for local exploration [33,34]. Furthermore, during iterative computation, the evolution rates of parameters across different dimensions in medical images vary. Parameters along the X- and Y-axes have a greater impact on similarity, while those along the Z-axis have a relatively smaller impact. Therefore, a slightly smaller *F* is set for this dimension compared to the others. Apart from the Z-axis, the evolution of the population is divided into two stages. Through extensive experimentation and comparison, it has been established that the *F* value is set to 0.5 for the first half stage and 0.8 for the latter half stage.

#### 3.5.3. Crossover

After the mutation operation, for each particle $X_{i,G}$ and its mutation vector $V_{i,G}$, a crossover operation is performed to generate the trial vector $U_{i,G}=\left\{u_{i,G}^{1},u_{i,G}^{2},\ldots ,u_{i,G}^{D}\right\}$. The most commonly used binomial crossover operation in PDE is depicted in Equation (11).

(11) $$u_{i,G}^{j}=\left\{\begin{array}{c} v_{i,G}^{j},\;if\left({rand}_{j}\left[0,1\right)\leq \mathrm{CR}\right)or\left(j=j_{rand}\right)\\ \;x_{i,G}^{j},\;otherwise \end{array}\right.$$

In Equation (11): j=1,2,…,D. The crossover probability *CR* is used to control the influence of parents in offspring generation, where *CR*∈ [0, 1). A higher value indicates less influence from the parents. Therefore, the evolution process is divided into two parts using 0.5 as the threshold: the first half stage is set with *CR* > 0.5, and the second half stage is set with *CR* < 0.5.

#### 3.5.4. Selection

Compute the objective function values for all trial vectors $u_{i,G}^{j}$, then compare the objective function value of each trial vector $u_{i,G}^{j}$ with the corresponding vector $x_{i,G}^{j}$ in the current population. If the objective function value of the trial vector $u_{i,G}^{j}$ is greater than that of the corresponding current vector, the trial vector $u_{i,G}^{j}$ will replace the respective current vector $x_{i,G}^{j}$, entering the next iteration of the population. The selection operation can be represented as follows.

(12) $$X_{i,G+1}=\left\{\begin{array}{c} U_{i,G},\;if\;f\left(U_{i,G}\right)>\left(X_{i,G}\right)\\ \;X_{i,G},\;otherwise \end{array}\right.$$

During the iterative optimization process, the CT images undergo pose parameter transformations generated randomly during population initialization. Each generated DRR image’s corresponding pose parameters are treated as a particle by the PDE algorithm. In each iteration, the similarity between each particle and the reference images in the frontal–lateral position is computed separately. The composite similarity, evaluated as *Sim*, measures the overall similarity of the images. Eventually, the particle with the highest similarity evaluation value represents the optimal particle. In the subsequent iteration of the population, the previous iteration’s optimal particle serves as the reference to generate new particles, aiming for higher similarity. When the maximum number of iterations is reached, the particle achieving the highest global fitness corresponds to the pose parameter combination with the highest similarity, and the corresponding DRR image represents the desired registration result. By refining the control parameters of population individuals and the mutation strategy, this algorithm exhibits efficient global optimization capabilities, preventing the algorithm from converging to local optima. Algorithm 2 is represented as follows.
**Algorithm 2.** Improved Differential Evolution.N: Population sizeD: Dimension of solution spacef(x): Objective function (to be minimized)$\left[x_{\min},x_{\max}\right]$: Search bounds$G_{\max}$: Maximum number of generationsCR: Crossover probabilityF: Scaling factor (0.5 for first half, 0.8 for second half)1: Initialize population $P=\{x_{i}|i=1,2,\ldots ,N\}$ randomly within bounds2: Compute fitness values $f_{i}=f\left(x_{i}\right)$ for all individuals3: Set initial optimal solution $x_{\mathrm{best}}=\arg\min\left(f_{i}\right)$, $f_{\mathrm{best}}=\min\left(f_{i}\right)$4: Set generation counter g=15: While $g\leq G_{\max}$ do:       Set F=0.5 if $g\leq G_{\max}/2$, else F=0.8       **for**
i=1:N
**do:**
           Randomly select 5 distinct indices $r_{1},r_{2},r_{3},r_{4},r_{5}\neq i$           Generate mutant vector:              $v_{i,G}=x_{r_{1},G}+F\cdot \left(x_{r_{2},G}-x_{r_{3},G}\right)+F\cdot \left(x_{r_{4},G}-x_{r_{5},G}\right)$           Generate trial vector $u_{i}$ via crossover:              for j=1:D do:                  if rand(0,1)≤CR or j=randint(1,D):                     $u_{i,j}=v_{i,j}$                  else:                     $u_{i,j}=x_{i,j}$           Evaluate fitness $f_{u}=f\left(u_{i}\right)$           if $f_{u}<f_{i}$, update $x_{i}=u_{i}$ and $f_{i}=f_{u}$       **End(for)**       Update $x_{\mathrm{best}}$ and $f_{\mathrm{best}}$ if new minimum found       Increment generation counter g=g+16: Return $x_{\mathrm{best}}$ and $f_{\mathrm{best}}$End

## 4. Experimental and Results

### 4.1. Dataset and Experimental Setup

The experiment utilized a real dataset obtained from a hospital, consisting of several sets of the spine, pelvis, and calcaneus medical images depicting various morphologies. X-ray medical images were acquired using frontal–lateral dual-plane imaging techniques for patients.

The hardware configuration used for the experiment consisted of an Intel(R) Core (TM) i5-4590 CPU @ 3.30GHz processor, an NVIDIA GeForce GTX 1060 6GB graphics card, and a PC running 64-bit Windows 10 Professional edition. The programming environment is Matlab R2020a, Visual Studio 2022.

### 4.2. Experiments with Intelligent Optimization Algorithms

From the CEC (Computational Experimental Competition) test suite [35,36], we selected six representative benchmark functions to evaluate the performance of the PDE algorithm in addressing complex optimization challenges. Detailed information about these benchmark functions is presented in Table 1. F1 to F3 represent single-mode benchmark functions designed to assess the algorithm’s optimization capability and convergence speed. F4 and F5 are multimodal benchmark functions that primarily examine the algorithm’s resilience against local optima. Lastly, F6 serves as a composite benchmark function.

**Figure 6:**
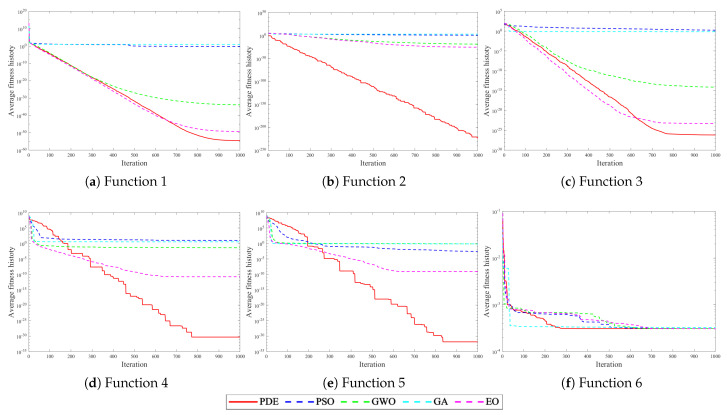
Convergence curve of benchmark functions.

The experiment set up the Particle Swarm Optimization (PSO), Grey Wolf Optimizer (GWO), Genetic Algorithm (GA), Equilibrium Optimizer (EO), and PDE algorithm to evaluate the optimization ability of the benchmark function. The maximum number of iterations is 1000, and all population sizes are set to 30. Figure 6 illustrates the function convergence curves of several optimization algorithms for the benchmark function.

In light of Figure 6, it is evident that the PSO, GWO, and GA exhibit poor performance for the single-modal function F1–F3, with subpar convergence speed and optimization-seeking ability. The EO and PDE, on the other hand, demonstrate the capability to approach the theoretical optimal values in single-mode functions. The PDE algorithm outperforms other algorithms in terms of both convergence speed and optimization accuracy on single-mode functions. For multimodal functions F4–F5, the PSO, GWO, and GA often get trapped in local optimality during the early and mid-term stages, leading to algorithm stagnation. The EO has a limited ability to escape from local extremes, and its convergence speed is notably slow. In conclusion, the PDE algorithm outshines other optimization algorithms in terms of convergence speed, optimization accuracy, and the ability to escape from local extremes in most test functions. It also exhibits robust global search capabilities.

To assess the efficiency of the PDE optimization algorithm, this experiment utilized three sets of spinal data with different morphologies. Comparative experiments in single-posture medical image registration were conducted, pitting the PDE algorithm against the Equilibrium Optimizer (EO) and Particle Swarm Optimization (PSO), with the aim of validating the efficiency of the proposed algorithm. The experiment was standardized with 50 iterations and 10 particles for each data group, and the average of 10 tests per group served as the registration result. Manual registration was considered the optimal result, and successful registration was defined as rotation differences less than 3° and translation differences less than 3 mm, accounting for minor errors in manual registration. The errors in the six degrees of freedom and the average registration time after using each algorithm are presented in Table 2.

The registration success rate of PDE reached 80.56%, surpassing the success rates of the EO (58.33%) and PSO (52.78%). Furthermore, the proposed algorithm reduced the average registration time by 4.14% compared to the other two optimization algorithms. The experimental results indicate that the proposed optimization algorithm demonstrates higher registration efficiency and stronger robustness.

### 4.3. Experiments on GPU Parallel Generation of DRR

In order to validate the accelerated impact of parallel DRR generation using the CUDA architecture, this experiment employed five sets of spinal data with varying morphologies. The PDE optimization algorithm was applied to traditional medical image registration with 50 iterations and 10 particles. The results, presented in Table 3, highlight a notable reduction of 43.71% in the average registration time when employing GPUs for multi-threaded parallel computation. These findings underscore the efficiency gains achieved through parallel DRR generation based on the CUDA framework, resulting in a substantial decrease in the time required for DRR generation.

### 4.4. Dual-Pose Registration Experiments

**Figure 7:**
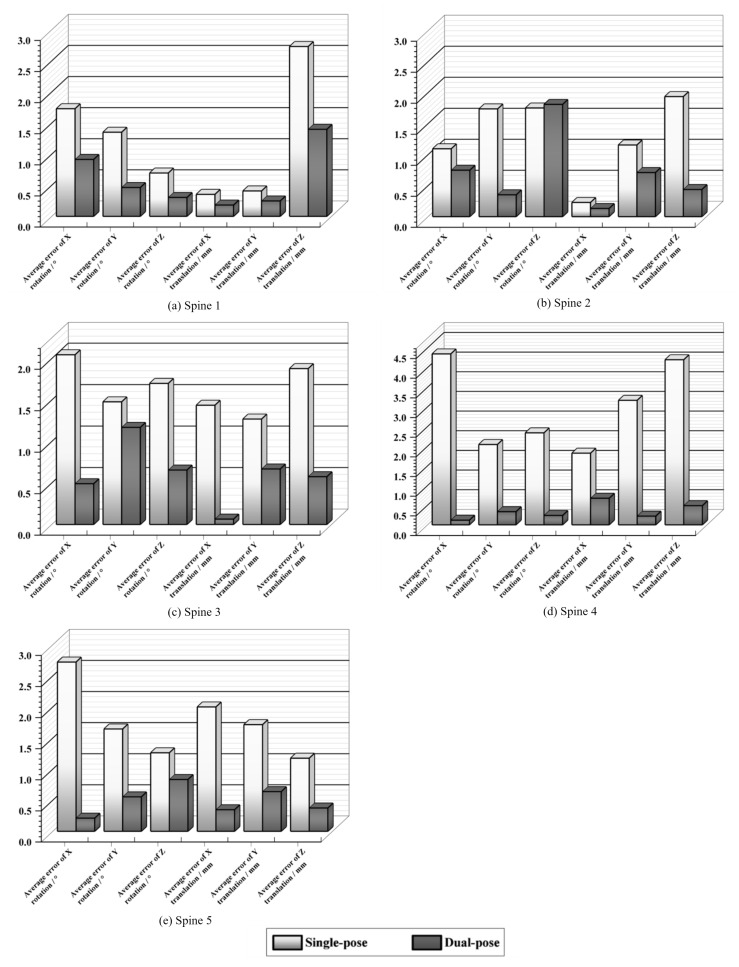
Comparative experiment of dual-pose strategy registration.

Utilizing the PDE optimization algorithm for single-posture registration, we conducted additional experiments for dual-pose registration using five sets of spinal data. The proposed optimization algorithm was configured with 50 iterations and 10 particles, and the average of 10 registrations per dataset was computed. The experimental results, illustrated in Figure 7a–e, showcase a successful rate of 81.33% achieved by the proposed algorithm. The pose parameters obtained from dual-pose registration exhibit a remarkable reduction of 67.04% in average rotation error and 71.84% in average translation error when compared to those obtained from single-posture registration. These results highlight that the proposed algorithm yields smaller registration errors and more precise registration results in dual-pose registration experiments conducted with five sets of spinal data.

### 4.5. Comparative Experiments of Registration Algorithms

To assess the accuracy of the proposed medical image registration algorithm, spine, pelvis, and calcaneus data were used for registration. Comparative experiments were conducted against three classical registration algorithms and two deep learning algorithms: MI, NCC, SSD, Siamese Network, and FaceNet. The proposed registration algorithm was set with 50 iterations and 10 particles, and each dataset underwent 10 experiments. The experimental results are presented in Table 4: the success rate of this algorithm is 85.40%, while the success rates of MI, NCC, SSD, Siamese Network, and FaceNet are 23.33%, 22.17%, 21.25%, 22.80%, and 22.93%, respectively. These results demonstrate that compared to other registration algorithms, this algorithm exhibits lower registration errors, higher registration efficiency, and significantly higher registration success rates.

**Figure 8:**
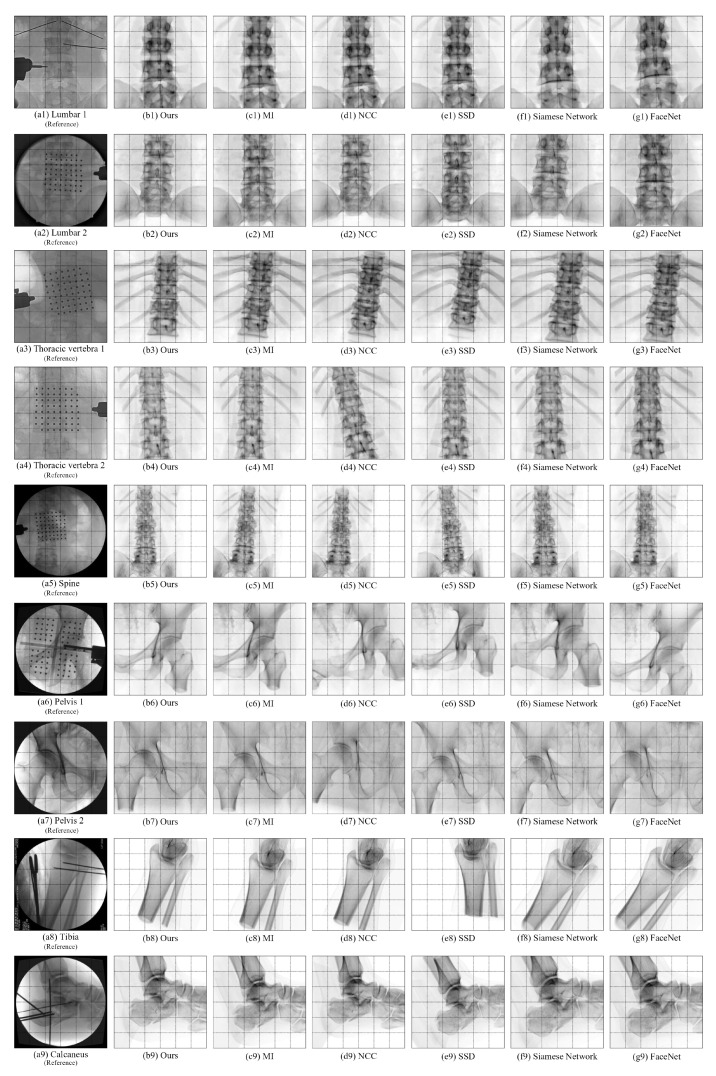
The registration results obtained by various algorithms.

The registration image obtained by this algorithm and the registration outcomes obtained by other registration algorithms are displayed in Figure 8. The figure shows that the algorithm proposed in this paper yields a registration result image with better translation distance and rotation angle accuracy in each degree of freedom than the other three registration algorithms, when compared to the original X-ray reference image.

## 5. Conclusions

Faced with the challenges of imprecise registration in traditional single-posture medical image registration, particularly for images with ambiguous features or prone to local optima, and significant errors in specific dimensions compared to other degrees of freedom, this paper introduces a novel dual-pose medical image registration algorithm based on an improved differential evolution approach. This method devises a composite measure to assess medical image similarity, employs GPU-accelerated Digitally Reconstructed Radiograph (DRR) technology, integrates dual-pose strategies to obtain frontal–lateral images to be registered, and subsequently utilizes PDE for iterative optimization. The result is a significant improvement in the similarity of DRR images, which serve as the final registration images.

The primary advantages of this algorithm are manifold. The utilization of dual-pose image registration incorporates richer projection view information and image-related details compared to single-posture images, enabling a more comprehensive analysis of six spatial degrees of freedom. This, in turn, reduces errors in rotation and translation, thereby enhancing the precision of medical image registration. The Phased Differential Evolution algorithm employed for optimization iteration improves the accuracy of registration results by refining the control parameters of population individuals and the mutation strategy, consequently augmenting the diversity of particle populations. Furthermore, the use of GPU-based parallel acceleration for image registration technology reduces the time overhead of GPU startup, thereby decreasing the DRR image generation time within the ray-casting algorithm and accelerating the overall execution efficiency of the algorithm.

While the proposed dual-pose medical image registration algorithm shows promising results in reducing registration errors, medical image registration remains a complex and challenging research domain. Future research could explore several directions: integrating deep learning techniques to enhance image preprocessing and contour extraction; investigating multimodal medical image registration, including 3D-3D registration; and exploring real-time medical image registration technology and its practical application in relevant clinical experiments.

## Figures and Tables

**Table 1:** Benchmark function.

Function	Search Scope	Theoretical Optimal Value
F1 = Schwefel 2.22	[−10,10]	0
F2 = Schwefel 1.2	[−100,100]	0
F3 = Schwefel 2.21	[−100,100]	0
F4 = Generalized Penalized 1	[−50,50]	0
F5 = Generalized Penalized 2	[−50,50]	0
F6 = Kowalik	[−5,5]	0.0003075

**Table 2:** Intelligent optimization algorithm registration experiments.

Bone Type	Algorithm	Average Error of X Rotation / °	Average Error of Y Rotation / °	Average Error of Z Rotation / °	Average Error of X Translation / mm	Average Error of Y Translation / mm	Average Error of Z Translation / mm	Average Registration Time / s
Spine 1	PSO	4.403	2.866	1.839	1.082	1.621	5.504	62.16
	EO	2.252	1.768	1.417	0.437	3.553	5.508	62.65
	**PDE (ours)**	**1.732**	**1.353**	**0.698**	**0.354**	**0.409**	**2.731**	**58.12**
Spine 2	PSO	5.913	2.115	3.984	0.671	1.670	5.086	64.61
	EO	3.784	2.039	2.564	0.489	1.487	3.047	65.93
	**PDE (ours)**	**1.095**	**1.739**	**1.753**	**0.229**	**1.156**	**1.937**	**63.72**
Spine 3	PSO	4.005	1.924	4.693	1.772	2.551	2.464	56.08
	EO	2.133	3.102	**1.257**	2.040	4.054	4.851	57.24
	**PDE (ours)**	**2.046**	**1.479**	1.701	**1.438**	**1.271**	**1.880**	**54.86**

**Table 3:** Experiments on GPU parallel generation of DRR.

Bone Type	Conventional Registration Time/ s	GPU Parallel Generation of DRR Registration Time/s	Speedup Ratio
Spine 1	58.12	31.00	1.8749
Spine 2	63.72	35.87	1.7762
Spine 3	54.86	30.19	1.8168
Spine 4	46.78	26.82	1.7445
Spine 5	64.42	38.29	1.6825

**Table 4:** Comparison with registration results of the state-of-the-art algorithms.

Algorithm	Bone Type	Average Error of X Rotation / °	Average Error of Y Rotation / °	Average Error of Z Rotation / °	Average Error of X Translation / mm	Average Error of Y Translation / mm	Average Error of Z Translation / mm	Average Registration Time / s
SSD	Lumbar 1	3.723	4.578	3.986	3.052	5.005	8.690	61.08
	Lumbar 2	3.690	2.468	2.726	2.267	16.194	8.639	
	Lumbar 3	2.598	5.953	1.463	1.581	5.183	10.356	
	Thoracic vertebra 1	13.035	8.614	3.915	8.009	1.223	8.464	
	Thoracic vertebra 2	5.951	1.160	1.792	4.799	1.286	10.555	
	Thoracic vertebra 3	5.142	1.712	3.950	5.484	8.544	15.533	
	Thoracic vertebra 4	4.620	4.316	2.114	16.006	1.751	14.582	
	Spine	10.234	1.740	1.617	12.165	3.361	11.208	
	Pelvis 1	11.077	5.547	2.511	3.837	2.663	6.221	
	Pelvis 2	4.744	2.534	1.651	2.875	3.402	14.787	
	Tibia	4.550	9.989	9.410	10.999	4.369	5.166	
	Calcaneus	2.750	4.440	12.936	1.675	7.716	13.753	
NCC	Lumbar 1	3.258	1.683	0.737	2.212	7.603	4.182	61.80
	Lumbar 2	1.267	1.759	1.422	1.091	2.098	4.027	
	Lumbar 3	3.898	1.172	0.560	2.163	7.645	5.649	
	Thoracic vertebra 1	9.279	4.383	0.833	7.519	5.109	6.742	
	Thoracic vertebra 2	6.402	4.854	4.558	2.816	6.941	4.325	
	Thoracic vertebra 3	4.497	1.401	3.986	5.094	7.301	9.764	
	Thoracic vertebra 4	9.643	1.157	1.281	9.412	**0.184**	7.363	
	Spine	7.598	**0.115**	1.024	8.685	9.761	11.525	
	Pelvis 1	3.774	9.057	8.350	9.205	11.867	3.560	
	Pelvis 2	8.481	4.837	6.500	8.868	3.868	4.276	
	Tibia	9.374	8.001	7.040	2.810	5.012	5.138	
	Calcaneus	**0.207**	6.988	2.897	2.899	1.881	15.986	
MI	Lumbar 1	5.976	2.148	2.408	1.310	7.922	5.484	59.41
	Lumbar 2	7.576	1.030	**0.467**	0.854	9.813	3.748	
	Lumbar 3	4.917	4.354	**0.276**	3.746	6.800	3.947	
	Thoracic vertebra 1	6.506	2.365	1.077	8.694	1.595	3.794	
	Thoracic vertebra 2	1.422	0.936	3.358	3.156	2.775	6.203	
	Thoracic vertebra 3	7.899	3.544	1.844	0.714	4.794	8.123	
	Thoracic vertebra 4	6.546	2.873	6.148	0.469	2.295	5.637	
	Spine	5.510	0.364	3.185	8.540	4.761	2.559	
	Pelvis 1	2.762	1.364	1.973	9.802	0.840	5.788	
	Pelvis 2	3.879	3.054	1.795	5.028	1.882	7.962	
	Tibia	7.399	6.420	3.047	1.071	8.159	5.883	
	Calcaneus	5.769	6.759	4.747	1.359	4.488	7.308	
Siamese Network [17]	Lumbar 1	12.321	1.156	2.452	2.390	4.013	9.817	85.90
	Lumbar 2	10.792	3.091	0.907	3.632	6.290	8.256	
	Lumbar 3	5.071	1.233	1.721	7.114	5.561	9.095	
	Thoracic vertebra 1	4.698	4.640	1.096	6.680	3.713	14.319	
	Thoracic vertebra 2	8.912	4.417	2.791	1.086	2.338	11.113	
	Thoracic vertebra 3	2.169	1.273	3.541	1.478	6.854	12.723	
	Thoracic vertebra 4	7.006	2.799	1.371	4.092	2.346	13.122	
	Spine	6.778	3.513	1.049	6.110	4.078	7.576	
	Pelvis 1	10.407	3.111	5.189	4.311	5.125	6.297	
	Pelvis 2	4.598	2.691	1.745	8.870	6.588	5.394	
	Tibia	7.967	9.225	3.046	**0.104**	10.223	11.749	
	Calcaneus	3.519	6.179	9.991	2.709	2.361	13.900	
FaceNet [19]	Lumbar 1	10.492	1.529	2.118	1.048	2.653	6.935	83.97
	Lumbar 2	12.086	2.478	1.754	1.487	8.517	14.821	
	Lumbar 3	11.723	5.121	1.790	5.137	6.051	8.622	
	Thoracic vertebra 1	6.208	2.650	1.207	3.987	7.017	11.059	
	Thoracic vertebra 2	13.220	3.072	1.253	2.421	1.769	10.890	
	Thoracic vertebra 3	9.350	2.131	6.797	0.339	10.311	12.198	
	Thoracic vertebra 4	8.563	4.131	3.147	8.157	3.768	8.893	
	Spine	9.116	2.266	1.576	10.652	7.985	7.920	
	Pelvis 1	4.897	3.762	8.898	9.023	9.158	9.114	
	Pelvis 2	9.766	2.048	4.638	8.412	3.551	4.645	
	Tibia	4.457	6.847	11.349	1.032	8.848	15.491	
	Calcaneus	1.551	5.217	9.441	2.222	5.412	10.160	
**Ours**	Lumbar 1	**0.916**	**0.465**	**0.304**	**0.182**	**0.248**	**1.400**	61.38
	Lumbar 2	**0.750**	**0.353**	1.812	**0.132**	**0.712**	**0.438**	
	Lumbar 3	**0.492**	**1.171**	0.657	**0.065**	**0.670**	**0.577**	
	Thoracic vertebra 1	**0.120**	**0.336**	**0.240**	**0.677**	**0.223**	**0.489**	
	Thoracic vertebra 2	**0.212**	**0.555**	**0.834**	**0.348**	**0.639**	**0.375**	
	Thoracic vertebra 3	**0.912**	**0.884**	**0.796**	**1.003**	**0.664**	**0.633**	
	Thoracic vertebra 4	**0.986**	**0.425**	**0.765**	**1.036**	1.674	**0.401**	
	Spine	**0.727**	0.271	**0.414**	**0.277**	**0.730**	**0.837**	
	Pelvis 1	**0.614**	**0.545**	**0.143**	**0.674**	**0.723**	**0.520**	
	Pelvis 2	**0.914**	**0.832**	**0.416**	**0.266**	**0.215**	**0.297**	
	Tibia	**0.628**	**0.956**	**0.883**	0.355	**0.359**	**0.496**	
	Calcaneus	0.294	**0.158**	**0.904**	**0.331**	**0.753**	**0.217**	

## Data Availability

Data are contained within the article.

## References

[1] Ou X.Y., Chen X., Xu X.N., Xie L.L., Chen X.F., Hong Z.Z., Bai H., Liu X.W., Chen Q.S., Li L. (2021). Recent Development in X-Ray Imaging Technology: Future and Challenges. Research.

[2] Qin C.X., Cao Z.G., Fan S.C., Wu Y.Q., Sun Y., Politis C., Wang C.L., Chen X.J. (2019). An oral and maxillofacial navigation system for implant placement with automatic identification of fiducial points. Int. J. Comput. Assist. Radiol. Surg..

[3] Liao R., Zhang L., Sun Y., Miao S., Chefd’Hotel C. (2013). A Review of Recent Advances in Registration Techniques Applied to Minimally Invasive Therapy. IEEE Trans. Multimedia.

[4] Naik R.R., Anitha H., Bhat S.N., Ampar N., Kundangar R. (2022). Realistic C-arm to PCT registration for vertebral localization in spine surgery. Med. Biol. Eng. Comput..

[5] Frysch R., Pfeiffer T., Rose G. (2021). A novel approach to 2D/3D registration of X-ray images using Grangeat’s relation. Med. Image Anal..

[6] Sotiras A., Davatzikos C., Paragios N. (2013). Deformable Medical Image Registration: A Survey. IEEE Trans. Med. Imaging.

[7] Gobbi D., Comeau R., Lee B., Peters T. Integration of intra-operative 3D ultrasound with pre-operative MRI for neurosurgical guidance. Proceedings of the 22nd Annual International Conference of the IEEE Engineering in Medicine and Biology Society (Cat. No. 00CH37143).

[8] Ban Y.X., Wang Y., Liu S., Yang B., Liu M.Z., Yin L.R., Zheng W.F. (2022). 2D/3D Multimode Medical Image Alignment Based on Spatial Histograms. Appl. Sci..

[9] Regodic M., Bardosi Z., Freysinger W. (2021). Automated fiducial marker detection and localization in volumetric computed tomography images: A three-step hybrid approach with deep learning. J. Med. Imaging.

[10] Yu W.M., Tannast M., Zheng G.Y. (2017). Non-rigid free-form 2D-3D registration using a B-spline-based statistical deformation model. Pattern Recognit..

[11] Kuppala K., Banda S., Barige T.R. (2020). An overview of deep learning methods for image registration with focus on feature-based approaches. Int. J. Image Data Fusion.

[12] Zhao J., Yang H., Ding Y. (2008). Medical image registration algorithm research based on mutual information similarity measure. Proc. SPIE.

[13] Tsai T.Y., Lu T.W., Chen C.M., Kuo M.Y., Hsu H.C. (2010). A volumetric model-based 2D to 3D registration method for measuring kinematics of natural knees with single-plane fluoroscopy. Med. Phys..

[14] Yan L., Wang Z.Q., Liu Y., Ye Z.Y. (2018). Generic and Automatic Markov Random Field-Based Registration for Multimodal Remote Sensing Image Using Grayscale and Gradient Information. Remote Sens..

[15] Damas S., Cordón O., Santamaría J. (2011). Medical Image Registration Using Evolutionary Computation: An Experimental Survey. IEEE Comput. Intell. Mag..

[16] Ma G.X., Ahmed N.K., Willke T.L., Yu P.S. (2021). Deep graph similarity learning: A survey. Data Min. Knowl. Discov..

[17] Li M.D., Chang K., Bearce B., Chang C.Y., Huang A.J., Campbell J., Brown J.M., Singh P., Hoebel K.V., Erdoğmuş D. (2020). Siamese neural networks for continuous disease severity evaluation and change detection in medical imaging. NPJ Digit. Med..

[18] Taigman Y., Ming Y., Ranzato M., Wolf L. DeepFace: Closing the Gap to Human-Level Performance in Face Verification. Proceedings of the IEEE Conference on Computer Vision and Pattern Recognition.

[19] McCauley J., Soleymani S., Williams B., Dando J., Nasrabadi N., Dawson J. Identical Twins as a facial similarity benchmark for human facial recognition. Proceedings of the 2021 International Conference of the Biometrics Special Interest Group (BIOSIG).

[20] Yunsheng B., Hao D., Yizhou S., Wei W. (2018). Convolutional set matching for graph similarity. arXiv.

[21] Spoerk J., Gendrin C., Weber C., Figl M., Pawiro S.A., Furtado H., Fabri D., Bloch C., Bergmann H., Gröller E. (2012). High-performance GPU-based rendering for real-time, rigid 2D/3D-image registration and motion prediction in radiation oncology. Z. Med. Phys..

[22] Banks S.A., Hodge W.A. (1996). Accurate measurement of three-dimensional knee replacement kinematics using single-plane fluoroscopy. IEEE Trans. Biomed. Eng..

[23] Akter M., Lambert A.J., Pickering M.R., Scarvell J.M., Smith P.N. A 2D-3D image registration algorithm using log-polar transforms for knee kinematic analysis. Proceedings of the 2012 International Conference on Digital Image Computing Techniques and Applications (DICTA).

[24] Shamshad F., Khan S., Zamir S.W., Khan M.H., Hayat M., Khan F.S., Fu H.Z. (2023). Transformers in medical imaging: A survey. Med. Image Anal..

[25] Almeida D.F., Astudillo P., Vandermeulen D. (2021). Three-dimensional image volumes from two-dimensional digitally reconstructed radiographs: A deep learning approach in lower limb CT scans. Med. Phys..

[26] Fluck O., Vetter C., Wein W., Kamen A., Preim B., Westermann R. (2011). A survey of medical image registration on graphics hardware. Comput. Methods Programs Biomed..

[27] Tahmasebi N., Boulanger P., Yun J.Y., Fallone G., Noga M., Punithakumar K. (2020). Real-Time Lung Tumor Tracking Using a CUDA Enabled Nonrigid Registration Algorithm for MRI. IEEE J. Transl. Eng. Health Med..

[28] Xi C., Li Z., Zheng Y. (2018). Deep similarity learning for multimodal medical images. Comput. Methods Biomech. Biomed. Eng. Imaging Vis..

[29] Chen X.X., Wang X.M., Zhang K., Fung K.M., Thai T.C., Moore K., Mannel R.S., Liu H., Zheng B., Qiu Y.C. (2022). Recent advances and clinical applications of deep learning in medical image analysis. Med. Image Anal..

[30] Nikolic M., Tuba E., Tuba M. Edge detection in medical ultrasound images using adjusted Canny edge detection algorithm. Proceedings of the 2016 24th Telecommunications Forum (TELFOR).

[31] Deng W., Shang S.F., Cai X., Zhao H.M., Song Y.J., Xu J.J. (2021). An improved differential evolution algorithm and its application in optimization problem. Soft Comput..

[32] Torres-Cerna C.E., Alanis A.Y., Poblete-Castro I., Bermejo-Jambrina M., Hernandez-Vargas E.A. A comparative study of differential evolution algorithms for parameter fitting procedures. Proceedings of the 2016 IEEE Congress on Evolutionary Computation (CEC).

[33] Gao S.C., Yu Y., Wang Y.R., Wang J.H., Cheng J.J., Zhou M.C. (2021). Chaotic Local Search-Based Differential Evolution Algorithms for Optimization. IEEE Trans. Syst. Man Cybern.-Syst..

[34] Bilal, Pant M., Zaheer H., Garcia-Hernandez L., Abraham A. (2020). Differential Evolution: A review of more than two decades of research. Eng. Appl. Artif. Intell..

[35] Yazdani D., Branke J., Omidvar M.N., Li X., Li C., Mavrovouniotis M., Nguyen T.T., Yang S., Yao X. (2021). IEEE CEC 2022 competition on dynamic optimization problems generated by generalized moving peaks benchmark. arXiv.

[36] Chen Q., Liu B., Zhang Q., Liang J., Suganthan P., Qu B. (2014). Problem Definitions and Evaluation Criteria for CEC 2015 Special Session on Bound Constrained Single-Objective Computationally Expensive Numerical Optimization.

